# Supplementary material for: COVID-19 vaccination attitudes and uptake: A sociocultural perspective focusing on parents and peers
Source: PLoS One. 2024 Jul 30;19(7):e0300771. doi: 10.1371/journal.pone.0300771 (PMC11288458; doi:10.1371/journal.pone.0300771)
Supplement: S1 File — (PDF) [file pone.0300771.s001.pdf]

Supporting Information

S1. Survey questions

1. Are you a student at a UK University or college? This includes undergraduate and postgraduate.
2. What was your age on your last birthday, in years?
3. How would you describe your gender?
4. Which country are you from?
5. What is your ethnicity?
6. Were you recommended to shield during the pandemic because of your own health? [Yes; No]
7. During the pandemic, have you been working in a care home and/or doing voluntary or paid carer work in someone's home and/or working as frontline health care staff (e.g., a nurse). [Yes; No]
8. Do you believe you have had, or currently have, coronavirus? [Definitely; Probably; Probably not; Definitely not; I don't know]
9. In December 2020, the UK Government announced that a coronavirus vaccination was available, and the rollout had begun. As far as you know, have you been offered the coronavirus vaccine? [Yes; No; I don't know; Prefer not to say]
10. Have you been vaccinated against COVID-19? [No; Yes, I've had one dose; Yes, I've had two OR MORE doses]
11. Now that a coronavirus vaccination is available, how likely is it that you will have one? Please select a number between 0 and 10, where 0 means ,"Extremely unlikely" and 10 means "Extremely likely" (If you do not know, please do not move the slider)
12. Do you have a mother or someone that you identify as a mother figure? [Yes; No; Prefer not to say]
13. Do you have a father or someone that you identify as a father figure? [Yes; No; Prefer not to say]
14. What is your relationship like with your mother? [Select a value between 1 = "Very negative" and 5 = "Very positive")
15. What is your relationship like with your father? [Select a value between 1 = "Very negative" and 5 = "Very positive")
16. In your opinion, a vaccination against COVID-19 is Effective (Select a value from 0 - Very ineffective to 10- Very effective)
17. In your opinion, a vaccination against COVID-19 is Safe (Select a value from 0 - Very unsafe to 10- Very safe)
18. In your opinion, a vaccination against COVID-19 is Important (Select a value from 0 - Very important to 10- Very important)
19. In your opinion, a vaccination against COVID-19 is Positive (Select a value from 0 - Very negative to 10- Very positive)
20. Has your mother been vaccinated against COVID-19? [No; Yes, she has had one dose; Yes, she has had two OR MORE doses]
21. Has your father been vaccinated against COVID-19? [No; Yes, he has had one dose; Yes, he has had two OR MORE doses]

22. Has your best friend been vaccinated against COVID-19? [No; Yes, they have had one dose; Yes, they have had two OR MORE doses]
23. Your mother thinks a vaccination against COVID-19 is Effective (Select a value from 0 - Very ineffective to 10- Very effective)
24. Your mother thinks a vaccination against COVID-19 is Safe (Select a value from 0 - Very unsafe to 10- Very safe)
25. Your mother thinks a vaccination against COVID-19 is Important (Select a value from 0 - Very important to 10- Very important)
26. Your mother thinks a vaccination against COVID-19 is Positive (Select a value from 0 - Very negative to 10- Very positive)
27. Your father thinks a vaccination against COVID-19 is Effective (Select a value from 0 - Very ineffective to 10- Very effective)
28. Your father thinks a vaccination against COVID-19 is Safe (Select a value from 0 - Very unsafe to 10- Very safe)
29. Your father thinks a vaccination against COVID-19 is Important (Select a value from 0 - Very important to 10- Very important)
30. Your father thinks a vaccination against COVID-19 is Positive (Select a value from 0 - Very negative to 10- Very positive)
31. Your best friend thinks a vaccination against COVID-19 is Effective (Select a value from 0 - Very ineffective to 10- Very effective)
32. Your best friend thinks a vaccination against COVID-19 is Safe (Select a value from 0 - Very unsafe to 10- Very safe)
33. Your best friend thinks a vaccination against COVID-19 is Important (Select a value from 0 - Very important to 10- Very important)
34. Your best friend thinks a vaccination against COVID-19 is Positive (Select a value from 0 - Very negative to 10- Very positive)
35. In your opinion, vaccinations in general are Effective (Select a value from 0 - Very ineffective to 10- Very effective)
36. In your opinion, vaccinations in general are Safe (Select a value from 0 - Very unsafe to 10- Very safe)
37. In your opinion, vaccinations in general are Important (Select a value from 0 - Very important to 10- Very important)
38. In your opinion, vaccinations in general are Positive (Select a value from 0 - Very negative to 10- Very positive)
